# An Early Description of Hematemesis in Persian Medicine

**DOI:** 10.18502/ijph.v49i10.4712

**Published:** 2020-10

**Authors:** Ali AMINIAN, Seyde Sedighe YOUSEFI

**Affiliations:** Department of Traditional Persian Medicine, Faculty of Medicine, Mazandaran University of Medical Sciences, Sari, Iran

## Dear Editor-in-Chief

Recent advances in gastrointestinal (GI) disorders, both in diagnosis and treatment, owes to long-term medical efforts and experiences, from the past to the present. Persian medicine (PM), as an important branch of Complementary and Alternative medicine (CAM), dating back to more than a thousand yaer history, with its outstanding scholars, especially during the golden age of the 9th to 12th centuries AD., played a major role in creating these scientific developments (1).

The upper gastrointestinal bleeding (UGIB) is one of the most important and common types of urgent GI manifestations, with high morbidity and mortality rates during hospitalization, which also imposes significant costs to the health system (2). Of course, this complication can be reduced significantly with timely diagnosis and appropriate treatment, although mortality rate still remains high, despite clinical progress (2). UGIB is described as an acute or chronic bleeding anywhere from the beginning of the esophagus to the end of the narrow intestine at the proximity of ligament of Treitz, which includes the esophagus, stomach, and duodenum (2, 3).

UGIB can be excreted through the mouth in the form of fresh red blood or coffee-ground clot which named “Hematemesis” or in the form of dark-colored feces through the colon known as “Melena” (2). Hematemesis, or the excretion of blood from the upper GI tract through the mouth, is one kind of UGIB bleeding that has been recognized and described by Persian medical philosophies more than a thousand year ago.

This description includes an important disease, explained in PM manuscripts, in the chapter of Gastric disorders by the title of “Gheye-al-Dam”, that means bloody vomiting, elsewhere from the esophagus or stomach up through the mouth (4–7). This definition seems to be equivalent to the current description of the Hematemesis in modern medicine. Same as current medicine, the causes of this disease are separately mentioned in Persian medicine, and is compared in Table 1, shows similarities between the two cases. According to PM point of view, hematemesis or 'Ghey- al-Dam', occurs due to different causes which the most important ones are: cracking or splitting of the upper GI vasculature because of various reasons, including rashes or ulcers in the GI tract, bursting of soft or hard mucosal swellings named ‘’ Ouram”, repeated retching or severe vomiting which leads to mucosal, vascular injury or bleeding and also consumption of drugs with hot temperament (Mizaj), which means drugs with such a quality that elevate internal body heat (4–7).

**Table 1:** Similarities between Persian and current medicine

***Causes of UGIB in Persian medicine***	***Causes of UGIB in Current medicine***
Rash, stomach or esophageal ulcer	Gastric ulcer, esophageal ulcer
Soft swelling or inflammation of the stomach or Esophagus	Gastritis, esophagitis
Hard swelling of the stomach or esophagus	Gastric or esophageal mass, malignancy
Dryness or excessive softness of the vascular wall	Vascular malformations
Bleeding post-repeated retching or severe vomiting	Mallory-Weiss syndrome
Painless splinting of the vessels	Duelafoy ulcer
Esophageal hemorrhoid	Esophageal varicose
Consumption such drugs with hot temperament	Drug side effects
Excretion of blood and waste materials from the liver to Stomach	Portal vein hypertension

In PM, therapeutic steps are similar to modern medicine, so that at first step, they used a compound syrup made up of boiled honey and water mixture named “Ma’a-al-Asal” for washing the bleeding area out of blood and clots (6), same as GI lavage in modern medicine (3), then in second step, with the purpose to control of bleeding, the PM philosophies recommended herbs with cold and dry temperament (Mizaj), that this quality causes to having astringent effect known as “Ghabiz” in PM, which is similar to vasoconstriction effect of drugs like epinephrine, that injects locally, and leads to control GI bleeding due to their ability in vascular contraction (2, 6).

Two of the most widely practiced herbs with astringent effect in PM, are Purslane (*Purtolaca oleraceae L*.) and Plantain (*Plantago major L*.) (4–6, 8). Understanding this fact and attending to the similarities between Persian medicine and modern medicine in diagnosis and treatments, and also further researches in hematemesis, may help the health system for better management, reduce the mortality rates and also treating costs.
